# The genome, transcriptome, and proteome of the nematode *Steinernema carpocapsae*: evolutionary signatures of a pathogenic lifestyle

**DOI:** 10.1038/srep37536

**Published:** 2016-11-23

**Authors:** Alejandra Rougon-Cardoso, Mitzi Flores-Ponce, Hilda Eréndira Ramos-Aboites, Christian Eduardo Martínez-Guerrero, You-Jin Hao, Luis Cunha, Jonathan Alejandro Rodríguez-Martínez, Cesaré Ovando-Vázquez, José Roberto Bermúdez-Barrientos, Cei Abreu-Goodger, Norberto Chavarría-Hernández, Nelson Simões, Rafael Montiel

**Affiliations:** 1 Laboratorio Nacional de Genómica para la Biodiversidad, Unidad de Genómica Avanzada, Centro de Investigación y de Estudios Avanzados del Instituto Politécnico Nacional. Km 9.6 Libramiento Norte Carretera Irapuato-León, C.P. 36821 Irapuato, Guanajuato, Mexico; 2 Laboratory of Agrogenomic Sciences, Universidad Nacional Autónoma de México (UNAM), ENES-León, 37684, León, Guanajuato, Mexico; 3 College of Life Science, ChongQing Normal University, ChongQing 401331, China; 4 Cardiff School of Biosciences, Cardiff University, Park Place, Sir Martin Evans Building, Museum Avenue, Cardiff, Wales CF10 3US, UK; 5 Cuerpo Académico de Biotecnología Agroalimentaria. Instituto de Ciencias Agropecuarias, Universidad Autónoma del Estado de Hidalgo. Av. Universidad Km 1, Rancho Universitario, Tulancingo de Bravo, Hidalgo, C.P. 43600, Mexico; 6 CIRN/Departamento de Biologia, Universidade dos Açores, Rua Mãe de Deus, 13. 9500-321 Ponta Delgada. S. Miguel-Açores, Portugal

## Abstract

The entomopathogenic nematode *Steinernema carpocapsae* has been widely used for the biological control of insect pests. It shares a symbiotic relationship with the bacterium *Xenorhabdus nematophila*, and is emerging as a genetic model to study symbiosis and pathogenesis. We obtained a high-quality draft of the nematode’s genome comprising 84,613,633 bp in 347 scaffolds, with an N50 of 1.24 Mb. To improve annotation, we sequenced both short and long RNA and conducted shotgun proteomic analyses. *S. carpocapsae* shares orthologous genes with other parasitic nematodes that are absent in the free-living nematode *C. elegans*, it has ncRNA families that are enriched in parasites, and expresses proteins putatively associated with parasitism and pathogenesis, suggesting an active role for the nematode during the pathogenic process. Host and parasites might engage in a co-evolutionary arms-race dynamic with genes participating in their interaction showing signatures of positive selection. Our analyses indicate that the consequence of this arms race is better characterized by positive selection altering specific functions instead of just increasing the number of positively selected genes, adding a new perspective to these co-evolutionary theories. We identified a protein, ATAD-3, that suggests a relevant role for mitochondrial function in the evolution and mechanisms of nematode parasitism.

Global losses due to pests can vary from about 26 to 80% depending on the type of crop^1^. Chemical pesticides are commonly used to fight this problem, however, they pose threats to humans, wildlife, and might have an adverse impact on soil fertility by killing beneficial microorganisms^2^. Other strategies rely on biological control agents, but their use is not generalized because of their limited efficiency when compared to pesticides. Genetic improvements are possible, especially when genomic information of the biological agent is available^3^,^4^. Entomopathogenic nematodes (EPNs) from the family of *Steinernematidae* have been commercialized in many countries as a biological insecticide for agricultural and horticultural crops and have attracted considerable attention because they are also potential models for symbiosis and pathogenesis^5^. One of the most well-known is *Steinernema carpocapsae* that shares a symbiotic relationship with the bacterium *Xenorhabdus nematophila*. Since it was thought that the bacteria were the main contributor to insect death, most research has focused on the pathogenic effect of the bacteria rather than the nematode^6^,^7^,^8^. Nevertheless, growing evidence suggests a more active role of the nematode in the pathogenic process^9^,^10^. In fact, a set of expanded gene families that are likely involved in parasitism were predicted in a recent genome analysis of *Steinernema* species^11^. Further genomic characterization will help to better understand the evolution and the function of these genomes in the symbiotic and pathogenic contexts. Parasitism is a common way of life among nematodes that has independently arisen at least 15 times during their evolution^12^. Particularly interesting are the phylogenetic associations between non-vertebrate and vertebrate parasites. The entomopathogenic Steinernematidae are phylogenetically related to Strongyloidoidae (Tylenchina; Panagrolaimomorpha), which infect mammals, suggesting a transition to vertebrate parasitism through host shifting^12^. The study of parasitism in *S. carpocapsae* should help to understand the origin and mechanisms of Strongyloidoids parasites, with implications for human health. For this study we produced a high-quality draft of the genome of *S. carpocapsae* strain Breton, and compared it with a recently published genome from a different strain of this species^11^. We further assessed the genetic signatures of its adaptation to a pathogenic lifestyle, and characterized the transcriptome by RNA-Seq, including both messenger RNA (mRNA), and small RNA (sRNA). We also present the most complete characterization to date of the proteome, generated by shotgun proteomics, two-dimensional gel electrophoresis (2DE) and SDS-PAGE. Additionally we conducted genome-wide scans for signatures of natural selection. We found several distinctive features related to pathogenesis through a comparison with both pathogenic and free-living nematodes.

## Results and Discussion

### Genome sequencing

Total DNA was extracted from isolated nuclei from a near isogenic line (~96% of estimated homozygosity) of *Steinernema carpocapsae* strain Breton. The use of isolated nuclei reduces the amount of symbiont and mitochondrial DNA, and the isogenic line was generated to avoid the acknowledged problems posed by heterozygosity for accurate genome assembly^12^. From one 454 shotgun library sequenced in three 454 FLX runs, we obtained 3,340,915 total reads with an average length of 357 bp. From one 454 paired-end library, with an insert size of 8 Kb, sequenced in two 454 FLX runs, we obtained 2,784,713 total reads with an average read length of 334 bp at each fragment end. From a SOLiD shotgun library sequenced in half a lane of SOLiD 5500xl, we obtained 24,942,584 reads of 75 bp (Table 1). By combining these long, paired-end, and short reads, we obtained a coverage of 32-fold, considering a genome size of ~110 Mb estimated by both flow cytometry and genome assembly. The final draft consists of 84,613,633 base pairs in 347 scaffolds, with an N50 of 1.24 Mega bases and with the largest scaffold of 8.7 Mb. This represents a notable improvement over a recently published genome that is more fragmented, with a much lower N50 (~0.3 Mb) and with the largest scaffold of only 1.7 Mb (Table 2). The average GC-content was of 45.67%, with 6.99% of repetitive sequences (Supplementary Table S1).

We assessed the completeness of the genome by analysing 248 ultra-conserved core eukaryotic genes^13^, obtaining 99.6% completeness considering partial genes and 99.2% for complete genes. These parameters indicated that our draft genome is of high quality, which gives us confidence in the genome annotation described below.

### Genome annotation

From the repetitive elements, we identified 1,702 distinct retrotransposon sequences representing at least eight families. Four were long interspersed element (LINE) groups, Cr1 being the most abundant, and 588 were short interspersed elements (SINEs), of which 432 belong to the tRNA-RTE family. We identified only two long terminal repeats (LTRs): *Gypsy* and *Pao*. We also identified eight families of DNA transposons, comprising 1,202 sequences, of which *hAT-Ac* was the most abundant with 388 elements, followed by *TcMar-Tc1, Merlin*, and the rolling-circle *Helitron* (327, 106, and 105 elements, respectively).

We collected RNA from pooled nematodes taken from all life cycle stages and subjected to various conditions (growing in larvae of two different insect species and on two different *in vitro* media, as described in Materials and Methods) in order to maximize the inclusion of condition-specific genes. We obtained 15,180,085 reads with an average length of 201 bp from an Illumina paired-end library on a MiSeq, and 92,231 reads with an average length of 288 bp from a 454 library on a partial 454 FLX + plate. After quality filtering, 94.93% of the reads mapped to the masked genome, suggesting a good reliability of the genome assembly. We performed genome-guided *de novo* assembly of the transcriptome that resulted in 21,457,711 bp of assembled transcripts (without introns). In order to identify protein-coding genes in the assembled genome, we assigned specific weights to different types of evidence to generate consensus gene calls (see Material and Methods). The current genome sequence and annotation is available at www.genomevolution.org (ID 33774), and at the NCBI GenBank (BioProject ID# 39853).

We identified 16,333 protein-coding genes with an average length of 1,257 bp, an average exon length of 222.37 bp, and an average of six exons per gene. We also identified 6,708 alternative transcripts and 5,725 truncated genes (defined as predicted protein-coding genes missing a start codon). We verified the protein expression of 3,773 predicted genes through mass spectrometry analysis (see below and Supplementary Table S2). The total number of predicted genes in this study is much lower than the previously predicted number of genes (28,313) for the strain “All” of the same species^11^. In the previous study, the heterozygosity was not reduced through the generation of an isogenic line, potentially negatively impacting on their genome assembly^12^. In addition, they performed gene prediction using Augustus^14^ with parameters optimized only for *Caenorhabditis elegans*. However, these species diverged ~280 million years ago^15^, making it difficult to accurately predict genes in *Steinernema* by solely using *C. elegans* gene models. To overcome this bias, we combined predictions using Hidden Markov Models (HMM) trained on *S. carpocapsae* gene structures with *ab initio* predictions, along with HMM homology-based predictions using *C. elegans* genes and *Brugia malayi* gene predictions (all the predictions were obtained with Augustus^14^). Although *B. malayi* has the same estimated time of divergence from *S. capocapsae* as *C. elegans*^15^, it is a parasite and therefore might share some homologous genes with *S. carpocapsae* that are not represented in *C. elegans*. However, the full strength of our approach is given by combining predictions using gene models from these species with *ab initio* predictions. When we used the same annotation strategy as in Dillman *et al*.^11^ using *C. elegans* models, we only obtained 14,188 protein coding genes. This is lower than the previous study, and also lower to the one we obtained with our combined strategy, in which the number of predicted genes probably increased due to the inclusion of *B. malayi* models, along with the *ab initio* predictions.

In summary, the use of an isogenic line (see Material and Methods), the combination of different sequencing platforms (Table 1), and an improved annotation strategy, resulted in a higher quality genome compared to a recent publication (Table 2). In any case, the studies used different strains of the nematode, and their goals were different, with Dillman *et al*.^10^ focusing on comparing their genome to that of other species of *Steinernema*. In our study, we obtained a higher quality genome, included analyses of the proteome and small RNAs, and performed a genome-wide scan of positive selection.

**Figure 1:**
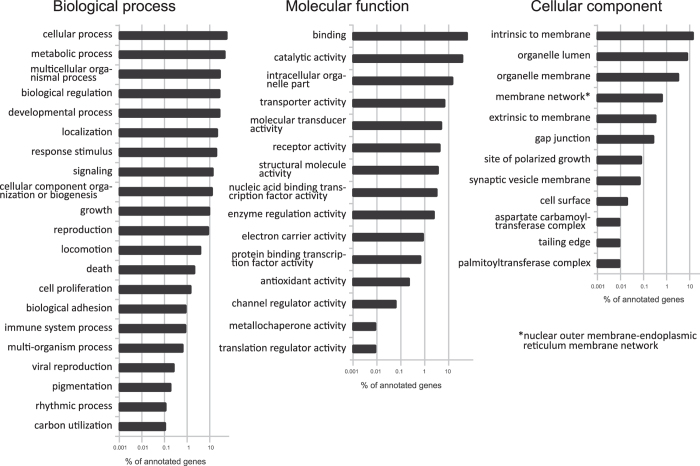
Enrichment analysis of GO terms in annotated sequences of *Steinernema carpocapsae,* in relation to those in *Caenorhabditis elegans*.

The most abundant GO terms in predicted genes are shown in Fig. 1. Our analysis revealed 135 enriched GO terms in *S. carpocapsae* when compared to those in *C. elegans* (Fisher’s exact test, FDR ≤ 0.05) (Supplementary Table S3). Many of these GO terms are involved in degradation, protein modification, binding and transport, and could be associated to parasitism (reviewed in ref. 16). At least 22 GO terms are also enriched in at least two other pathogenic worms, but not in the free-living nematode *Pristionchus pacificus* (Table 3). Supplementary Table S4 shows the abundance of the different protein families in 10 different nematode genomes compared with the 30 most abundant families in *S. carpocapsae* (Supplementary Fig. S1). Most of the expanded families (16 out of 20) identified previously in the “All” strain of *S. carpocapsae*^11^ are also overrepresented in our strain (Breton). In addition, Peptidase S1 is also overrepresented in *S. carpocapsae* and other parasites (*Bursaphelenchus xylophilus, Meloidogyne hapla,* and *M. incognita*) compared to *C. elegans*. Integrase, enoyl-acyl-carrier-protein reductase (ENR) (IPR014358), retrotransposon pao, and pimelyl-acyl-carrier protein methyl ester esterase PFAM domains, are also overrepresented in some parasites, including *S. carpocapsae*.

**Figure 2:**
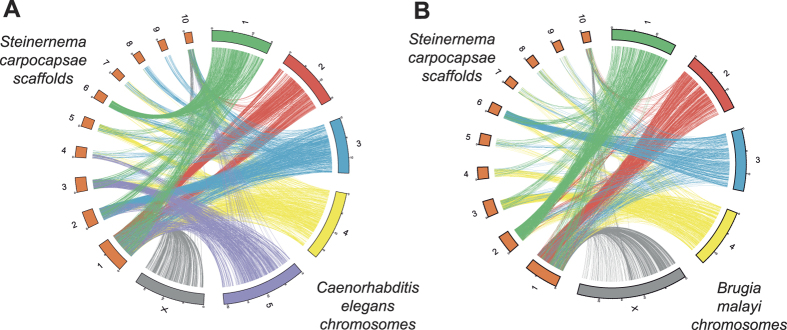
Schematic representation of shared sequences between *Steinernema carpocasae* and (**A**) *Caenorhabidits elegans*, or (**B**) *Brugia malayi*, both based in HSPs (E-value < 1e-6).

Since *C. elegans* and *S. carpocapsae* are phylogenetically distant from one another, we found no evident macro-synteny. However, there are genes located in single chromosomes of *C. elegans* that match genes located in single scaffolds of *S. carpocapsae*. A similar result was obtained in a comparison with the *Brugia malayi* genome (version WS253) (Fig. 2 and Table 4), even though this genome is not of the same quality as that of *C. elegans*. This reinforces the idea that the use of *B. malayi* gene models in the annotation strategy is at least as good as the use of *C. elegans* models.

Beyond the protein-coding potential of the genome, we predicted non-coding RNA (ncRNA) using a variety of tools (see Materials and Methods), identifying 1,097 tRNAs, 40 rRNAs (15 5S rRNA, 1 5.8S rRNA and 24 8S rRNA), 38 micro-RNA hairpins and 146 other ncRNAs. Using the same annotation pipeline, we compared the abundance of each ncRNA family in parasitic (*Ascaris suum, Bursaphelenchus xylophilus, Brugia malayi, S. carpocasae, M. incognita, M. hapla and Heterorhabditis bacteriophora*) and free-living (*Panagrellus redivivus, Pristionchus pacificus, C. remanei and C. elegans*) nematodes (Supplementary Table S5). By comparing the average number of elements in each family between parasitic and free-living nematodes, we derived a simple metric to decide if a family had a tendency to be enriched in one of the two lifestyles (see Materials and Methods). The families enriched in parasitic nematodes are, ACEA_U3 (a snoRNA), SeC (a tRNA), mir-100/mir-10, mir-227, mir-2b, mir-2444, and mir-4455 (microRNAs), all of which have at least twice the number of elements on average in the parasites (Supplementary Fig. S2). Although the correlation between these families and parasitism needs to be further investigated, this is a first indication that these ncRNA families might have a functional role in the pathogenic lifestyle.

To complement these bioinformatic predictions we performed small RNA-seq of *S. carpocapsae* with and without induction with insect hemolymph. We obtained a total of 42.8 million reads from 6 libraries. Less than 10% of the cleaned reads failed to map to the genome (see Materials and Methods), another indication that the genome assembly is very complete. We used two of the most popular tools to annotate known and novel microRNAs using small RNA sequencing data: miRDeep^17^ and ShortStack^18^. Both tools coincided in predicting 100 miRNAs, while miRDeep predicted an additional 162, and ShortStack 25 more, giving a total of 287 miRNA hairpins, each with a potential 5′ and 3′ mature product (Supplementary Table S6). These predictions followed an expected length distribution, with a dominant peak centred at 22 nucleotides. Of the sRNA sequencing reads of 20–24 nucleotides that mapped to the genome, 83% overlapped with the 287 miRNA hairpins (Supplementary Fig. S3). Interestingly, we detected a large number of novel miRNA genes, since only 25 out of the 287 predicted miRNA hairpins correspond to known miRNAs according to homology searches. Although the majority of the conserved miRNAs tend to have high expression in our experiments, half of the 20 most highly expressed miRNA predictions correspond to novel sequences (Supplementary Table S7). This confirms the great diversity of ncRNA genes that are species or lineage specific, and highlights the importance of using experimental data when annotating genomes, particularly for species that are distant to well annotated model organisms.

We were also interested to see if any of the microRNAs that we detected changed their expression in response to insect haemolymph. None of the miRNAs showed a significant decrease in expression, but five increased their expression after hemolymph induction (Supplementary Table S7 and Supplementary Fig. S4). These miRNAs were miR-84–3p, miR-84-5p, miR-31-3p, let-7-5p and Cluster_21397_3p (a new prediction with no similarity to known miRNAs). Interestingly, the induced miRNAs included miR-84 and let-7, members of the let-7 family of miRNAs that are important players during development. In *Caenorhabditis elegans*, double mutants of miR-84 and miR-48 (another let-7 family member) show a delayed moulting phenotype and accumulate a double cuticle^19^. This is interesting because *S. carpocapsae* infective juveniles have a double cuticle that is lost upon entering the insect host^8^. The up-regulation of miR-84 and let-7 could thus be involved in the moulting process, triggered by the contact with insect hemolymph.

### Differentially expressed proteins

**Figure 3:**
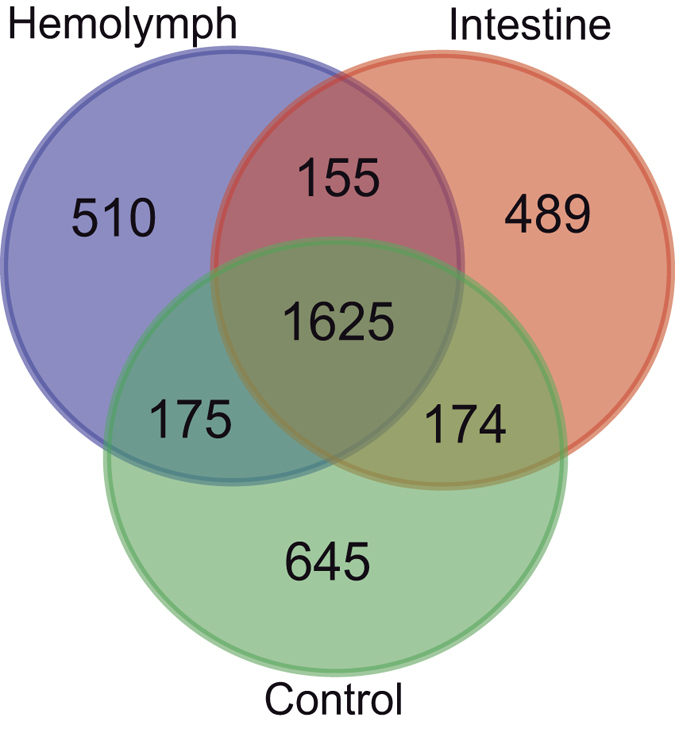
Non-redundant soluble proteins expressed after induction of *Steinernema carpocapsae* IJs with insect intestines, hemolymph or non-induced control.

During infection, the nematodes first invade the insect intestine and then cross the intestinal wall by expressing putative effectors that facilitate parasite penetration to the hemocoel, where they continue to counteract insect defences^10^,^20^. Therefore, we were interested in comparing the soluble proteins from Infective Juveniles (IJs) induced with either insect intestines or insect hemolymph, against non-induced controls. We opted for a detection strategy combining shotgun proteomics strategy, with two-dimensional electrophoresis (2DE), and SDS-PAGE, that resulted in the identification of 7,527 proteins. By eliminating duplicates (proteins that were detected more than once), we obtained 3,773 non-redundant proteins (Supplementary Table S2). Among the non-redundant proteins, 1,625 were expressed in the three conditions, 155 were only expressed under both hemolymph and intestine induction, and 349 were expressed in the control and one other condition. In addition, 489 proteins were exclusively expressed in nematodes induced with intestine, 510 in those induced with hemolymph, and 645 in the non-induced controls (Fig. 3). This suggests that specific activities occur at different stages during the pathogenic process. Proteins expressed specifically in the induced conditions, were associated with functional categories (GO terms) using Blast2GO (Supplementary Figs S5 and S6). We found four GO terms enriched among the 489 proteins expressed exclusively in the intestine-induced sample, 10 GO terms in the 510 proteins of the hemolymph-induced sample, and 8 GO terms in the 1,154 combined proteins from both induction conditions (Fisher’s exact test, p < 0.05); in all cases in relation to the untreated control (Supplementary Table S8). One of the differentially expressed proteins was a transthyretin-like protein (TLP). Unlike transthyretins, which are known for transporting thyroxine and related molecules, the function of TLPs is not well understood^21^. They seem to have a role in the uricase reaction pathway as 5-hydroxyisourate hydrolases^22^. Their abundance in parasitic nematodes and more specifically their expression during parasitic stages, suggests an involvement of TLPs in parasitism^22^,^23^. We also found other differentially expressed peptides in the parasitic stages of *S. carpocapsae* that, being found in other parasites, could be involved in the infective processes (Supplementary Table S2).

### Proteases and excretory/secretory proteins

We found all types of proteases in the genome of *S. carpocapsae.* Proportions of most of them (Aspartic, Cysteine, and Threonine) are similar to the proportions found in free-living (*C. elegans*), necromeric (*P. pacificus*), and parasitic (*B. malayi, Strongyloides ratti*) nematodes. However, the amount of serine proteases in *S. carpocapsae* and *P. pacificus* genomes is higher than in other nematodes (39% and 33.2% of all proteases, respectively); while in *S. carpocapsae* the percentage of metalloproteases, although high (31%), is the lowest in the comparison (the highest is in the *Strongyloides ratti* genome with 52%).

Excretory/secretory (ES) products are complex mixtures of hundreds of different proteins that are thought to have important roles in the life cycle of a parasite and during host-parasite interactions^24^. A total of 1,421 putative excreted proteins were predicted in the genome (see Methods), including various families of proteases, protease inhibitors, cuticular collagens, and C-type lectins, as well as putative signalling molecules such as warthog, ground and ground-like proteins (Supplementary Table S9). Some ES proteins are predicted to be involved in immuno-evasion; such as collagen^25^, whereas others play crucial roles in the suppression of host immune responses by mimicking host molecules, such as C-lectin^26^,^27^. Furthermore, C-lectins have been found to be upregulated in *Ancylostoma ceylanicum* at the onset of heavy blood feeding from the host^28^. We also found three putative copies of parasitic stage specific protein 1, a protein without known domains, which is present in several parasitic nematodes and is expressed during the transition to the parasitic lifestyle in *Haemonchus contortus*^29^. Other interesting putative secreted peptides in the *S. carpocapsae* genome were lipases, saposins, and transthyretin-like protein, all of which are expressed during early parasitic stages in *H. contortus*^29^. Serine proteases, including Sc-SP-1 and Sc-SP-3, can mediate the invasion or apoptosis of host cells^10^,^20^. Astacin metalloprotease is one of the effector molecules involved in tissue invasion of parasitic nematodes^30^. The family of papain-type aspartic and cysteine proteases are thought to have the same role in invertebrate digestion as trypsin in vertebrates^31^. Therefore, it is possible that the dependence of *S. carpocapsae* on aspartic protease activities is related to the digestion of nutrients. Cysteine proteases are involved in digestive processes or moulting and cuticle renewal in free-living and parasitic nematodes^32^,^33^.

### Orthologous proteins

We compared 7,724 orthologous groups of proteins among several species with different lifestyles. We found 318 orthologous groups that are absent in non-pathogenic species (*Caenorhabditis angaria, C. remanei, C. briggsae, C. japonica, C. elegans, and Pristionchus pacificus*) but present in *S. carpocapsae* and in at least another pathogenic nematode (Supplementary Table S10). The annotations of these orthologues revealed enrichment of protein functions with possible associations with parasitism, such as serine proteases and other terms related to degradation and binding (Table 5). We also found 134 additional groups from the orthoMCL-DB (version 5) database that are present in the genome of *S. carpocapsae* but not in the other tested species (Supplementary Table S11).

### Positive selection

Because of the co-evolutionary arms-race relationship between hosts and their pathogens, genes involved in their interaction are expected to evolve under positive selection^34^, potentially resulting in specific genomic signatures associated with their lifestyles^35^. We used the branch-sites test of positive selection^36^,^37^ to analyse 2,034 orthologous genes in three species of Clade IV nematodes (as defined in ref. 38). This test is based on a maximum likelihood estimation of the nucleotide nonsynonymous and synonymous substitutions rates. The ratio of nonsynonymous to synonymous rates (ω) can be used to identify purifying selection (ω < 1), neutral evolution (ω = 1), or positive selection (ω > 1), assessing the significance with a Likelihood Ratio Test (LRT)^36^. We found 83 genes with sites evolving under positive selection (ω > 1, LRT, p < 0.05; 14 of which had an FDR < 0.1) in *S. carpocapsae* (Table 6). Among the 83 genes, 23 GO terms were significantly enriched (Supplementary Table S12) when compared to the genes with no sites under positive selection (1,951 genes) (Fisher’s exact test, p < 0.01). Although we found more genes (95) with sites evolving under positive selection in the free-living nematode *Panagrellus redivivus*, there were no enriched GO terms among them (even with an alpha value of 0.05), indicating that the consequence of an arms-race relationship is better characterized by positive selection preferentially altering genes of specific functions than just increasing the number of positively selected genes. Although we would need to increase the number of analysed genes to increase the power of these analyses, the initial results suggest a new perspective to the co-evolutionary arms-race theories.

### Phylogenetic analysis

**Figure 4:**
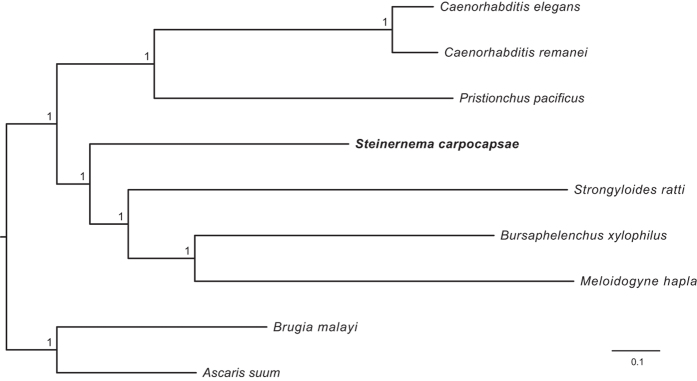
Bayesian phylogenetic tree reconstructed from the concatenated alignment of 245 orthologous proteins of nine nematode species. Numbers in branches are posterior probabilities.

To explore the phylogenetic relationships of *S. carpocapsae*, we reconstructed a phylogeny using 245 proteins from strictly 1-1 orthologous genes from nine nematode species. According to Blaxter *et al*.^38^, *Steinernema* is phylogenetically closer to *Strongyloides* than to *Caenorhabditis,* as inferred from a tree reconstructed using the small subunit ribosomal DNA (18S) sequences from 53 nematode species. A similar result was obtained in a more extensive analysis using 339 18S sequences^39^. However, Montiel *et al*.^40^ found *Steinernema* to be closer to *Caenorhabditis* than to *Strongyloides* using complete mtDNA sequences. Although this discrepancy may result from differential reproductive strategies and/or differential selective pressures acting on nuclear and mitochondrial genes^40^, an analysis of large subunit ribosomal DNA sequences (28S) also showed *Steinernema* to be closer to *Caenorhabditis*^41^. Defining these relationships is relevant because if *Steinernema* is phylogenetically closer to *Strongyloides*, it could be used as a more general model for parasitism, with implications for human health. *Steinernema* is more tractable than *Strongyloides* because it does not require a vertebrate host to reproduce in the laboratory. Our new phylogenetic analysis supports *Steinernema* being closer to *Strongyloides* than to *Caenorhabditis* (Fig. 4). In addition, its basal position in relation to *Strongyloides*, gives support to the hypothesis that this vertebrate parasite originated by host shifting from an entomopathogenic ancestor^12^, in this case *Steinernema*.

### Gene functions enriched at several levels

To assess how the pathogenic lifestyle is affecting specific gene functions at different levels, we compared the GO terms enriched in the genome of *S. carpocapsae* when compared to *C. elegans* (Supplementary Table S3), with those in differentially expressed proteins due to hemolymph or intestine induction (Supplementary Table S8), and with those in genes with putative sites evolving under positive selection (Supplementary Table S12). One GO term was enriched in the genome and in differentially expressed proteins (transcription factor activity – sequence-specific DNA binding); and two GO terms in both the genome and genes under positive selection (macromolecular complex and ribonucleoprotein complex). No GO terms were shared between the three analyses. However, one protein, ATAD-3 (ATPase family AAA domain-containing protein 3), is associated with 33 enriched GO terms in the annotated sequences, and two additional enriched GO terms from genes with evidence of positive selection (Supplementary Table S13). This protein is differentially expressed in nematodes induced with insect tissues (intestines) and presents amino acid sites evolving under positive selection. Functions or proteins shared between these analyses might reveal relevant effects of the pathogenic lifestyle in the genome. A deeper analysis of positive selection (i.e. including more orthologous genes or conducting population genetic analyses) could expand the number of shared genes, which should be good candidates for further studies. In this case we have identified a putative homolog of *C. elegans*’ ATAD-3. Its deficiency in *C. elegans* causes early larval arrest, gonadal dysfunction, and embryonic lethality. It is also associated with defects in organellar structure and mtDNA depletion^42^,^43^, suggesting that ATAD-3 is important for increased mitochondrial activity during the transition to later larval stages^42^. *S. carpocapsae* needs to go through developmental changes to establish itself in the insect body during the pathogenic process, which might explain the relevance of this, and probably other mitochondria-related genes, in nematode parasitism. For example, the defective mitochondrial respiration family member protein 1, with functions in regulation of growth rate, was differentially expressed in nematodes induced with insect tissues (hemolymph) and presented evidence of positive selection. Mitochondria have been identified as important contributors to the virulence of fungal pathogens^44^, and it has been previously hypothesised that differential selective constraints in mitochondrial genes might explain discrepancies between nuclear and mitochondrial gene phylogenies in nematodes^40^. In addition, depletion of ATAD-3 in *C. elegans* resulted in reduced intestinal fat storage^42^, and it would be interesting to explore if fat metabolism might also be relevant in nematode parasitism.

## Conclusion

Our genomic analyses of *S. carpocapsae* confirm a role in pathogenicity beyond simply vectoring the symbiotic bacteria. *S. carpocapsae* shares orthologous genes with other parasitic nematodes that are absent in the free-living nematode *C. elegans*, it encodes ncRNA families that are enriched in parasites, and presents putative proteins associated with functions related to parasitism and pathogenesis. Until now, the best examples of positive selection in genes related to host-pathogen interactions were pathogen effectors and genes of the host immune and defence systems^34^. Our analyses indicate that positive selection can also alter genes belonging to other functional categories, such as metabolism and development, adding a new aspect to the arms-race co-evolutionary theories. Through a comprehensive analysis, we identified a protein, ATAD-3, suggesting a relevant role for mitochondria during the evolution of nematode parasitism that warrants further investigation. We provide additional evidence for the phylogenetically relatedness of *S. carpocapsae* to *Strongyloides*, making this high-quality genome valuable for comparative studies with potential implications for human health. Our genome also represents a useful resource to aid ongoing efforts towards the genetic improvement of entomopathogens as biological control agents as well as to better understand host-parasite interactions in nematodes.

## Materials and Methods

### Organisms, maintenance and storage

*Steinernema carpocapsae* strain Breton was obtained from Nelson Simões, and cultured using *in vitro* methods. Nematodes were grown using a modified protocol for mass production in artificial medium according to ref. 45, as well as in small-scale, on plates containing Fortified Lipid Agar (FLA) prepared with 1.6% TSB (nutrient broth), 1% vegetable oil, 1.2% bacteriological agar, and 5% yeast extract (modified from ref. 46). A near isogenic line of *S. carpocapsae* strain Breton was generated by reproduction of single couples of brother and sister for 12 generations (F12). This produces ~96% homozygosis^47^.

### DNA isolation, sequencing and quality control

Total genomic DNA was isolated from the nuclei^48^ using the phenol/chloroform extraction protocol described by Sambrook *et al*.^49^. Total DNA yield and integrity was measured with a 2100 Bioanalyzer (Agilent) using an Expert High Sensitivity DNA chip. Three high-quality libraries for Next Generation Sequencing (NGS) were prepared following manufacturer’s instructions. One shotgun 454 library was sequenced in three 454 FLX runs. One 454 paired-end library with 8-kb inserts was sequenced in two 454 FLX runs. Finally, a SOLiD shotgun library was tagged and sequenced, along with a different library (from other organisms), in a lane of SOLiD 5500xl, equivalent to half a lane of SOLiD sequencing. Low-quality sequences, base-calling duplicates and adapters were removed from all the sequence data (see below).

### Genomic assembly and filtering

All DNA-sequence reads were filtered to remove contamination of the endosymbiotic bacteria *Xenorhabdus nematophila* (Xn). A genomic dataset was created by adding the published genome of Xn strain ATCC 19061 to the unpublished genome of the Xn strain isolated from the *S. carpocapsae* strain Breton nematodes, produced in our laboratory. The dataset was used for contamination screening and filtering using GS Assembler 2.7.

Raw standard flowgram format (sff) files coming from 454 platforms were assembled using GS Assembler 2.7 with the default trimming parameters. Basespace reads coming from the SOLiD platform using the Exact Call Chemistry Module (that allows conversion from colour to basespace) were filtered to remove PCR clonal repeats as well as reads with ambiguous bases. Subsequently, sequences were filtered based on Phred quality values. Bases below Phred18 were removed from 3′ ends and only reads longer than 20 bp were kept. Filtered data were assembled into contigs using GS Assembler 2.7, and joined into scaffolds using the paired-end data.

GC-content was estimated from the scaffolds using 10-kb non-overlapping sliding windows, and GC-bias was assessed based on a frequency distribution of these data. To evaluate the completeness of the genome assembly, we followed two strategies. RNA-seq sequences representing all different stages and diverse culture conditions of *S. carpocapsae* were mapped to the final assembly using Newbler (GS Reference Mapper v.2.7). In addition, we analysed the completeness of 248 ultra-conserved core eukaryotic genes^13^. We expect a complete genome will contain a higher number of complete ultra-conserved genes.

### Estimation of genome size

Genome size was estimated from the genomic assembly using GS Assembler 2.7 and corroborated through flow cytometry of the isolated cellular nuclei of *S. carpocapsae* using the nuclei of *C. elegans* strain N2 (genome size approx. 100 Mb^50^) as a size control. Nuclei were stained with CyStain® UV Ploidy (Partec 05-5001), and fluorescence was detected at λ ≤ 420 nm and quantified using a PARTECPAII (Partec, Germany) flow cytometer with a mercury lamp (100 W UV light).

### Assessment of repeat content

Following genome assembly, repeats were identified using a combination of homology-based comparisons (using RepeatMasker^51^) and a *de novo* approach (using RepeatModeler^52^).

### Annotation of non-coding RNA

Covariance models from Rfam^53^ were used to scan the genomes using Infernal software^54^. In addition, tRNAs were predicted using tRNAscan-SE^55^ and rRNAs were predicted using RNAmmer^56^. Finally, microRNA precursor sequences (miRNA hairpins) were located using MapMi^57^, using all mature miRNA sequences from miRBase 21^58^ as input. MapMi results were filtered selecting only microRNA precursor sequences with score ≥30. Results from Rfam, tRNAscan-SE, RNAmmer and MapMi were processed within R (R: A Language and Environment for Statistical Computing; http://www.r-project.org), using ‘GenomicFeatures’ and ‘rtracklayer’ packages^59^,^60^.

To compare ncRNA families present in parasitic (*Ascaris suum, Bursaphelenchus xylophilus, Brugia malayi, S. carpocasae, Meloidogyne incognita, M. hapla, and Heterorhabditis bacteriophora*) and free-living (*Panagrellus redivivus, Pristionchus pacificus, Caenorhabditis remanei, and C. elegans*) nematodes, the average number of genes belonging to each ncRNA family, were calculated for each group. Families with at least twice the average number of genes in the parasitic compared to free-living group were selected.

### RNA isolation, sequencing and assembly

Total RNA was extracted from a pool of individuals from all lifecycle stages (eggs at different stages, L1, L2, L3, IJ, L4, and adults), cultured *in vivo* infecting *Galleria mellonella* and *Tenebrio molitor* larvae as described^61^, as well as all lifecycle stages cultured *in vitro* using the methods described in the Organisms, maintenance and storage section^45^,^46^. The final pool consisted of approximately 3 mg of individuals from each stage/condition. RNA was extracted using TRIzol (Invitrogen) according to the manufacturer’s instructions with an additional step using Qiagen RNAeasy Mini Elute Clean up columns and buffers to clean and concentrate the RNA.

An Illumina paired-end library and a 454 library were generated for RNA-seq, which were run on a full plate of MiSeq and a partial plate of a 454 FLX+, respectively. RNA-seq reads were quality filtered and mapped to the repeat-masked genome using Newbler gsmapper. Read alignments were provided to Trinity^62^ (r2013-02-25) as a coordinate-sorted bam file. Trinity was used to assemble the aligned reads. The Trinity-reconstructed transcripts were aligned and assembled using the PASA2^63^ (r20130425 beta) pipeline.

For small RNA (sRNA) sequencing, nematodes were induced for 2 hour with hemolymph of *Galleria mellonella* and with buffer as control^64^. Nematodes were grinded under liquid nitrogen and RNA was extracted with Trizol according to the manufacturer’s instructions. Six sRNA-Seq tagged libraries were prepared from three replicates of each condition, which were run in an Illumina HiSeq lane.

### Processing small RNA sequencing results

All sRNA-Seq libraries were 3′-adaptor trimmed using the reaper tool from Kraken^65^. After trimming, reads between 18 and 36 nucleotides were mapped to the genome using ShortStack 3.3^18^, setting the maximum number of mismatches to 1, no stich, multi-mappers guided by unique-mappers and removing reads that mapped to more than 101 locations. The raw and processed sRNA-Seq results were deposited in GEO (http://www.ncbi.nlm.nih.gov/geo), under accession GSE85256.

### Predicting expressed microRNA loci with miRDeep and ShortStack

When using ShortStack to annotate, we set the Dicer minimum and maximum size parameters to 18 and 36, minimum alignment coverage (mincov) to 5 and maximum distance to merge clusters (pad) to 50. To improve the predicted microRNA producing loci, we used MirDeep2^17^. The mapper.pl and miRDeep2.pl modules were used to identify known and novel microRNAs. Reads between 18 and 36 nucleotides were used, with a maximum number of mismatches of 1, and 101 maximum number of locations for multi-mapping reads. The minimum alignment coverage (-a) was set to 5, the maximum number of precursors to analyze was set to 1000, and all the mature sequences from miRBase 21 were provided. Although both programs take small RNA sequencing reads mapped to a genome to predict microRNA loci, they produce slightly different results. They coincided in predicting 100 miRNAs, while miRDeep predicted an additional 162 and ShortStack 25 more.

### Differential expression analysis of microRNAs

To focus the differential expression analysis on miRNAs, only reads in the 20–24 nucleotide length range were considered. After mapping these to the genome, on average 83% fell within miRNA hairpin and 63% within mature miRNA coordinates. For miRNA quantification, the featureCounts function of the Rsubread R package^66^ was used, asking for a minimum overlap of one nucleotide to any of the mature miRNA annotations. For differential expression analysis, the edgeR package was used^67^. miRNAs with less than 3 counts-per-million in at least 3 libraries were removed, leaving 302 out of the 574 annotated mature miRNAs. The trimmed mean of M-values was chosen as normalization method^68^. Genewise data dispersion was estimated with the function estimateGLMTagwiseDisp, which uses an empirical Bayes strategy^67^. Differentially expressed miRNAs were determined with a generalized linear model and gene-wise likelihood ratio tests. A False Discovery Rate threshold of 0.1 was selected to consider a miRNA to be significantly differentially expressed. According to this threshold, five miRNAs were overexpressed in response to hemolymph treatment and none were down regulated. Cluster_19164 was identified as miR-84 by a manual sequence search on the miRBase website^58^.

### Gene prediction and synteny

The *S. carpocapsae* protein-coding gene set was inferred using *de novo*, homology- and evidence-based approaches (Supplementary Fig. S7). *De novo* gene prediction was performed on a repeat-masked genome using Augustus^14^. Training models were generated using hints from a compilation of *S. carpocapsae* gene structures (CEGMA^13^ [v2.4.010312] predictions, PASA assemblies from our RNA-seq data, and 2,269 publicly available ESTs from GeneBank). The homology-based prediction was conducted with Augustus algorithms for *C. elegans* and *Brugia malayi*. Synteny was assessed on scaffolds >1 Mb using pairwise alignments with E-value < 10^−6^ and homologous regions were visualized using CIRCOS^69^. Macro-synteny was analysed using the SynFind and SynMap tools from CoGe^70^,^71^.

### Functional annotation of coding genes

Following the prediction of the protein-coding gene set, we conducted high-stringency BLASTp homology searches (E-value ≤ 10^−5^) against the NCBI non-redundant protein database. Functional annotation was performed using Blast2GO^72^. Gene ontology categories were summarized and standardized to level 2 and level 3 terms, defined using the GOslim hierarchy^73^. For the secretome prediction, the signal peptide was predicted by SignalP 4.0^74^ and Phobious^75^ employing both Hidden Markov Models and Neural Networks. Proteins were then filtered for the presence of transmembrane regions using THMMN^76^ and Phobious^75^. Subcellular localizations were identified using TargetP (≥95% specificity)^77^ and WolfPSORT^78^ (score ≥30). Proteases and protease inhibitors were identified by homology searches to the MEROPS database^79^.

### Orthologous proteins

Genes from different nematode species (*Caenorhabditis angaria, C. briggsae, C. elegans, C. japonica, C. remanei, Pristionchus pacificus, Ascaris suum, Brugia malayi, Bursaphelenchus xylophilus, Heterorhabditis bacteriophora, Haemonchus contortus, Loa loa, Meloidogyne hapla, M. incognita, Onchocerca volvulus, Panagrellus redivivus, S. carpocasae, Strongyloides ratti, Trichinella spiralis, and Wuchereria bancrofti*) were assigned to OrthoMCL^80^ orthologous groups, and the presence or absence of groups was compared among the different species using ad hoc scripts from scriptome (http://archive.sysbio.harvard.edu/csb/resources/computational/scriptome/UNIX/).

### Additional bioinformatics analyses and use of software

Data analysis was conducted in a UNIX environment or Microsoft Excel 2007 using standard commands. Bioinformatic scripts required to facilitate data analysis were designed using bash, GNU coreutils, Perl, and Python.

### Proteomic analysis

#### Sample preparation

Nematodes were induced as described^64^ with slight modifications. A pool of approximately 25,000 nematodes were induced with either hemolymph or intestine of *Galleria mellonella*. To obtain hemolymph we grinded *G. mellonella* larvae in liquid nitrogen, added a volume of cold Tyrode’s solution (NaCl 0.8%, KCl 0.02%, CaCl_2_ 0.02%, MgCl_2_, 0.02%, NaH_2_PO_4_, 0.005%, NaHCO_3_, 0.1%, and glucose, 0.1%) and sonicated at a frequency of 20 kHz. Then we centrifuged at 1700 rcf for 15 min at 4 °C, obtaining three phases, of which the middle one corresponded to hemolymph. Intestines were dissected from insect larvae, collected on a watch glass and rinsed several times with sterile saline solution (NaCl 0.8%) to eliminate any trace of hemolymph or other remains. Nematode infecting juveniles (IJs) were superficially disinfected with 2% sodium hypochlorite during 10 min, and rinsed three times with sterilized water. According to our experience, this treatment is enough to kill all surface bacteria and fungi from the nematodes while keeping them viable. Washed nematodes were then transferred to a 90 × 15 mm Petri dish containing 7 ml of Tyrode’s solution with 10% of *G. mellonella* hemolymph (v/v) or 10% of intestines (w/v), and 1% Nalidixic acid, to avoid contamination. Previous experience in the Simões lab has shown that 10% of hemolymph is needed to induce recovery of the IJs and to allow them to complete their life cycle. The same concentration of intestines was used as a first approximation to understand the effects of insect intestines on protein expression. Different pools of nematodes were incubated under agitation (40 rpm) at 25 °C for 1, 2, 4 and 8 hours, and analysed separately. To determine these time points we followed the infection process *in vivo*, by conducting dissections at regular intervals, to understand the kinetics of the infection. We observed that after entering the intestine, it took the nematodes one hour to start traversing the intestine wall and at two hours, most of them were in the hemocoel. We included two additional time points to capture proteins expressed lately in the infection process; stopping at 8 hours because at this point the nematodes start to release the symbiotic bacteria^81^. Nematodes without induction were used as a negative control. Nematodes were grinded in liquid nitrogen and suspended in lysis buffer (7 M urea, 2 M thiourea, 3% CHAPS) with protease inhibitor mix GE and sonicated at a frequency of 20 kHz. After centrifugation (1 min at 16,000 rcf) and filtering (0.45 μm Millex-HV PVDF, Millipore), the supernatant was precipitated with the 2D Clean-up kit (GE Healthcare), resuspended in DeStreak solution (GE Healthcare Cat. No. 17600318), and quantified with the Bradford Method^82^ (BioRad Protein Assay Dye Cat. No. 500-0006).

#### SDS-PAGE and 2D electrophoresis

Protein samples (80 μg) were loaded onto the SDS-PAGE and ran at 100 V in a vertical Mini-PROTEAN Tetra cell BioRad. Gels were stained with Coomassie blue. Isoelectric focusing was performed using GE Healthcare Immobiline strips pH 3-10 and DryStrip pH 4–7, in both cases of 7 cm of length. Isoelectric focusing was performed on a Multiphor II (GE Healthcare) using the conditions recommended by the manufacturer. The second dimension was run on 13% polyacrylamide gels.

#### Shotgun proteomics

Two hundred μl of the total protein extract was fractionated by isoelectric focusing in an IEF ZOOM® Fractionator system (Life Technologies) using the protocol described by the manufacturer. The protein pellet was passed through a reduction/alkylation process with urea (6 M), dithiothreitol (DTT) at a final concentration of 5 mM, and iodoacetamide (IAA) at a final concentration of 15 mM. Peptides were digested with trypsin (Promega) overnight at 37 °C and desalted with a Macro Spin Column (Nest Group). Thirty μg of protein from each fraction were then analyzed by LC-MS/MS in the Proteomics Facility of the UC Davis Genome Center. A Thermo Scientific Q Exactive Orbitrap MS spectrometer was used in conjunction with a Proxeon Easy-nLC II HPLC (Thermo Scientific) and a source Proxeon nanospray using a column 100 micron × 25 mm Magic C18 5U 100 Å reverse phase. The MS/MS spectra were acquired using the TOP15 method following the equipment manufacturer’s instructions. All analyses were run in duplicates, including treatment samples and controls. ProteinPilot (v4.5), Mascot (v2.4), MaxQuant (v1.3.0.5), and Sequest (v1.3) software were used to identify peptides and proteins in each sample (see software references in Supplementary Table S2). In all cases, a tolerance in the mass measurement of 50 ppm in MS mode and 0.5 Da for MS/MS ions was used, with a significant threshold set to p < 0.05 and a confidence value ≥95%, with the exception of MaxQuant, in which the peptide mass tolerance was of 20 ppm, the fragment mass tolerance of 0.5 Da, and the confidence value was ≥99%. Modifications allowed were carbamidomethylation C (fixed), deamination NQ (variable), and oxidation M (variable).

Proteins detected in at least one sample replica and undetected in the two control replicates were considered as differentially expressed proteins. Proteins detected in at least one of the control replicates, but undetected in the two sample replicates were considered differentially suppressed proteins. Annotation and functional enrichment of differentially expressed proteins were performed with Blast2GO^72^. The Fisher Exact Test was used to compare the GO terms identified under the different induction conditions (hemolymph or gut) with the nematodes without induction.

### Phylogenetic analysis

Protein sequences of all organisms were downloaded from WormBase (ftp.wormbase.org release WS241 29-Nov-2013). Orthologous genes for nine species were identified with OrthoMCL (v5)^80^. A total of 245 orthologous proteins were aligned with MUSCLE (v3.8.31)^83^. Phylogenetically informative blocks were recovered with Gblocks^84^ and the best-fit evolutionary model for each aligned protein was predicted by ProtTest^85^. MrBayes^86^ was used for phylogenetic reconstruction using concatenated alignments. Partitions were created by grouping proteins according to their best-fit model, i.e. each partition contained all the proteins evolving under the same model. A mixed model was applied to each partition, with different G, I, and F parameters and unlinking the model between partitions. To check for convergence, two runs with four chains each were performed. The analysis was run for 1,000,000 generations, and a burn-in of 25% was used. *Brugia malayi* and *Ascaris suum* were used to root the tree because these species were the most phylogenetically basal of the nine nematode species in the phylogenies obtained by both Blaxter *et al*.^38^ and Nadler *et al*.^41^.

### Analysis of positive selection

We used protein-coding genes from *Panagrellus redivivus, Strongyloides ratti* and *Steinernema carpocapsae*, all nematodes from phylogenetic clade IV, according to Blaxter *et al*.^38^. All nucleic and amino acid sequences, except for *S. carpocapsae*, were downloaded from WormBase (ftp.wormbase.org release WS241 29-Nov-2013). Orthologues proteins obtained with OrthoMCL (v5)^80^ were aligned with ClustalW2 (v2.1)^87^. After selecting phylogenetically informative sites with Gblocks^84^, and estimating the best-fit model with ProtTest^85^, we reconstructed a consensus phylogenetic tree with PhyML (v.3.0)^88^. A nucleotide alignment based on the complete amino acid alignment was obtained with RevTrans (v1.4)^89^ to preserve codon homology. The tree and the nucleotide alignments of each orthologous gene were used to assess signatures of natural selection with Codeml from the PAML package (v.4.6)^90^, using the Branch-site model to identify genes with sites under positive selection. Annotation and functional enrichment in genes with positively selected sites were performed with Blast2GO^72^.

## Additional Information

**How to cite this article**: Rougon-Cardoso, A. *et al*. The genome, transcriptome, and proteome of the nematode *Steinernema carpocapsae*: evolutionary signatures of a pathogenic lifestyle. *Sci. Rep.* **6**, 37536; doi: 10.1038/srep37536 (2016).

**Publisher's note:** Springer Nature remains neutral with regard to jurisdictional claims in published maps and institutional affiliations.

## Supplementary Material

Supplementary Figures S1–S7

Supplementary Table S1

Supplementary Table S2

Supplementary Table S3

Supplementary Table S4

Supplementary Table S5

Supplementary Table S6

Supplementary Table S7

Supplementary Table S8

Supplementary Table S9

Supplementary Table S10

Supplementary Table S11

Supplementary Table S12

Supplementary Table S13

## Figures and Tables

**Table 1:** Summary of sequencing data from *Steinernema carpocapsae* strain Breton, compared to the sequencing data of the strain All^11^.

Strain	Source	Library type	Platform	No. of runs	No. of reads (Millions)	Average read length (bp)	Insert size (bp)
Breton	DNA	Shotgun	454 GS FLX	3	33.41	357	—
		Paired-end	454 GS FLX	2	27.85	334	8000
		Shotgun	SOLiD 5500xl	~Half lane	24.94	75	—
	RNA	Shotgun cDNA	454 GS FLX +	Half plate	0.09	288	—
		Paired-end cDNA	Illumina MiSeq	1	15.18	201	—
	sRNA	Shotgun	Illumina HiSeq 2500	1 lane (6 tagged libraries)	42.81	51	—
All	DNA	Paired-end	Illumina Genome Analyzer IIx	1	85.76	75	400
		Paired-end	Illumina Genome Analyzer IIx	1	103.09	100	350
		Paired-end	Illumina HiSeq 2000	1	131.59	100	1800
	RNA	Paired-end cDNA	Illumina Genome Analyzer IIx	4 (each from a different developmental stage)	260.86	75	200

**Table 2:** Summary statistics of assembly and annotation of the genome of *Steinernema carpocapsae* strain Breton, compared to the assembly of the strain All^11^.

Strain	Breton	All
Sequencing depth	32X	330X
Estimated genome size in megabases (GSAssembler 2.7)	111.3	85.6
(Flow Cytometry)	~110	Not determined
Number of scaffolds	347	1,578
Total number of base pairs within assembled scaffolds	84,613,633	86,127,942*
N50 Scaffold length (bp)	1,245,171	299,566
Largest scaffold (bp)	8,793,593	1,722,607
GC content of whole genome (%)	45.67	45.53
Repetitive sequences (%)	6.99	7.46
Proportion of genome that is coding (exonic) (%)	19.72	38.8*
Proportion of genome that is transcribed (exons + introns) (%)	42.09	50.31*
Number of putative coding genes	16,333	28,313
Number of non-coding RNAs	1,317	Not determined
Mean gene size (bp)	2,681	2,030
Mean coding sequence length per gene (bp)	1,257	1,046*
Average exon number per gene	6	5
Average gene exon length (bp)	222.37	212
Average gene intron length (bp)	145.44	194
GC content in coding regions (%)	52.49	51.86
Functionally annotated genes (according to BLAST2GO default parameters)	10,395 (63.6%)	Not determined

^*^Calculated from version PRJNA202318.WBPS6 obtained from www.wormbase.org.

**Table 3:** Enriched GO terms in the genome of *Steinernema carpocapsae* and in at least two other pathogenic species but not in the free-living nematode *Pristionchus pacificus*, as compared to the free-living nematode *Caenorhabditis elegans*.

GO-ID	Term	Sc	As	Bm	Bx	Di	Hb	Ll	Mh	Mi	Ov	Sr	Ts	#Sp
GO:0005524	ATP binding	↑	↑	↑	↑	↑	↑	↑	↑	↑	↑	↑	↑	12
GO:0003743	translation initiation factor activity	↑	↑	↑		↑	↑	↑	↑	↑	↑	↑	↑	11
GO:0003964	RNA-directed DNA polymerase activity	↑	↑	↑	↑	↑	↑		↑	↑	↑	↑	↑	11
GO:0006278	RNA-dependent DNA replication	↑	↑	↑	↑	↑	↑		↑	↑	↑	↑	↑	11
GO:0015074	DNA integration	↑		↑	↑	↑	↑	↑	↑	↑	↑	↑	↑	11
GO:0034754	cellular hormone metabolic process	↑	↑			↑		↑	↑	↑	↑	↑	↑	9
GO:0019915	lipid storage	↑	↑		↑	↑		↑	↑				↑	7
GO:0008284	positive regulation of cell proliferation	↑	↑				↑	↑			↑	↑		6
GO:0004190	aspartic-type endopeptidase activity	↑			↑				↑	↑		↑	↑	6
GO:0006446	regulation of translational initiation	↑	↑	↑				↑						4
GO:0006744	ubiquinone biosynthetic process	↑		↑		↑					↑			4
GO:0008270	zinc ion binding	↑		↑						↑			↑	4
GO:0045333	cellular respiration	↑		↑		↑					↑			4
GO:0055114	oxidation-reduction process	↑					↑		↑	↑				4
GO:0006913	nucleocytoplasmic transport	↑			↑							↑		3
GO:0015992	proton transport	↑			↑				↑					3
GO:0046339	diacylglycerol metabolic process	↑			↑					↑				3
GO:0004180	carboxypeptidase activity	↑				↑						↑		3
GO:0006886	intracellular protein transport	↑					↑					↑		3
GO:0009792	embryo development ending in birth or egg hatching	↑					↑					↑		3
GO:0006821	chloride transport	↑							↑			↑		3
GO:0004252	serine-type endopeptidase activity	↑								↑			↑	3

*Sc, Steinernema carpocapsae*; As, *Ascaris summ*; Bm, *Brugia malayi*; Bx, *Bursaphelenchus xylophilus*; Di, *Dirofilaria immitis*; Hb, *Heterorhabditis bacteriophora*; Ll, *Loa loa*; Mh, *Meloidogyne hapla*; Mi, *M. incognita*; Ov, *Onchocerca volvulus*; Sr, *Strongyloides ratti*; Ts, *Taenia solium*; ↑, enriched term in that species (and in *S. carpocapsae* but not in *P. pacificus*) as compared with *C. elegans*.

**Table 4:** Percentage of genes located in single chromosomes (Chr) of *Caenorhabidtis elegans* (Ce, above) or *Brugia malayi* (Bm, below) that match genes located in single scaffolds of *Steinernema carpocapsae*.

*Steinernema carpocapsae*											
	Scaffold	01	02	03	04	05	06	07	08	09	10
	Total genes	1761	864	638	333	332	540	406	409	273	399
Ce (%)	Chr1	5.91	5.90	4.23	1.20	4.82	55.37	3.69	6.60	5.13	6.77
	Chr2	30.44	7.06	6.90	4.20	23.49	7.96	6.65	5.87	6.59	9.27
	Chr3	5.79	37.73	3.76	5.11	5.42	5.19	5.91	7.09	6.96	9.02
	Chr4	10.96	8.22	7.99	4.20	24.40	7.04	7.14	8.80	44.32	8.02
	Chr5	6.93	6.71	39.34	23.12	5.12	6.30	5.42	5.87	5.13	9.77
	ChrX	26.69	4.05	5.33	4.20	4.22	5.19	18.23	17.85	5.49	27.82
	Without match	13.29	30.32	32.45	57.96	32.53	12.96	52.96	47.92	26.37	29.32
Bm (%)	Chr1	4.09	37.96	4.08	3.30	3.92	5.19	2.96	1.71	5.86	3.26
	Chr2	27.77	5.90	4.86	4.20	21.39	4.81	5.42	6.60	5.49	4.76
	Chr3	3.92	2.78	2.35	1.80	3.61	55.93	2.22	2.69	2.93	2.76
	Chr4	2.90	5.90	37.30	18.92	3.01	5.00	3.45	3.91	4.03	3.26
	ChrX	26.35	7.41	9.40	6.91	19.28	8.52	22.17	21.27	36.63	35.09
	Without match	34.98	40.05	42.01	64.86	48.80	20.56	63.79	63.81	45.05	50.88

**Table 5:** Enrichment of Gene Ontology (GO) terms of orthologs abscent in non-pathogenic nematodes (*Caenorhabditis angaria, C. remanei, C. briggsae, C. japonica, C. elegans, and Pristionchus pacificus*) and present in *S. carpocapsae* and at least another parasitic nematode compared to *C. elegans* GO terms.

GO-ID	Term	Category	FDR	P-Value
GO:0045449	regulation of transcription, DNA-dependent	P	4.30E-16	8.08E-20
GO:0004252	serine-type endopeptidase activity	F	7.73E-15	2.90E-18
GO:0005667	transcription factor complex	C	8.03E-12	4.52E-15
GO:0008236	serine-type peptidase activity	F	1.25E-11	1.17E-14
GO:0017171	serine hydrolase activity	F	1.25E-11	1.17E-14
GO:0045941	positive regulation of transcription, DNA-dependent	P	2.16E-05	2.44E-08
GO:0004175	endopeptidase activity	F	1.78E-04	2.34E-07
GO:0043234	protein complex	C	3.18E-03	5.09E-06
GO:0005515	protein binding	F	3.18E-03	5.37E-06
GO:0045935	positive regulation of nucleobase-containing compound metabolic process	P	4.95E-03	1.11E-05
GO:0051254	positive regulation of RNA metabolic process	P	4.95E-03	1.11E-05
GO:0051173	positive regulation of nitrogen compound metabolic process	P	4.95E-03	1.11E-05
GO:0008233	peptidase activity	F	8.30E-03	2.02E-05
GO:0003713	transcription coactivator activity	F	8.41E-03	2.21E-05
GO:0010628	positive regulation of gene expression	P	1.00E-02	2.98E-05
GO:0043170	macromolecule metabolic process	P	1.00E-02	3.02E-05
GO:0070011	peptidase activity, acting on L-amino acid peptides	F	1.01E-02	3.40E-05
GO:0006508	proteolysis	P	1.01E-02	3.42E-05
GO:0010557	positive regulation of macromolecule biosynthetic process	P	3.65E-02	1.49E-04
GO:0045298	tubulin complex	C	3.65E-02	1.58E-04
GO:0033202	DNA helicase complex	C	3.65E-02	1.58E-04
GO:0031011	Ino80 complex	C	3.65E-02	1.58E-04
GO:0097346	INO80-type complex	C	3.65E-02	1.58E-04

**Table 6:** Genes with sites evolving under positive selection in *Steinernema carpocapsae* (Sc), *Panagrellus redivivus* (Pr), and *Strongyloides ratti* (Sr).

Orthologue genes analysed by the Branch-site test	N = 2,034		
Tested branch	Sc	Pr	Sr
Genes with sites under positive selection (ω > 1, LRT, p < 0.05)	83 (4.08%)	95 (4.67%)	8 (0.39%)
Average proportion of sites under positive selection per gene (s.d.)	6.49% (0.067)	7.47% (0.093)	17.31% (0.187)
