# Supplementary material for: The genome, transcriptome, and proteome of the nematode Steinernema carpocapsae: evolutionary signatures of a pathogenic lifestyle
Source: Sci Rep. 2016 Nov 23;6:37536. doi: 10.1038/srep37536 (PMC5120318; doi:10.1038/srep37536)

# The genome, transcriptome, and proteome of the nematode *Steinernema carpocapsae*: evolutionary signatures of a pathogenic lifestyle

Rougon-Cardoso A., Flores-Ponce, M., Ramos-Aboites, H.E., Martinez-Guerrero, C.E., Hao, Y-J., Cunha, L., Rodríguez-Martinez J.A., Ovando-Vázquez, C., Bermúdez-Barrientos, J.R., Abreu-Goodger, C., Chavarria-Hernández, N., Simões, N., Montiel, R.

## SUPPLEMENTARY FIGURES

**Supplementary Figure S1**. Graphic representation of the abundance of the different protein families (PFAM domains) in 10 different nematode genomes compared with the top 40 abundant families in *S. carpocapsae*.

**Supplementary Figure S2**. Enrichment of ncRNA families according to nematode life-styles. Heatmap showing the number of ncRNA producing loci according to nematode life-style (parasitic and free-living). The maximum number of loci is trimmed at 8, for display purposes.

**Supplementary Figure S3**. Small RNA length distribution of ShortStack and miRDeep2 predictions. A stacked barplot shows the total number of reads of each length that mapped to the genome, and the fraction of these that overlap with miRNA loci predicted by miRDeep2 (orange), ShortStack (purple) or both methods (green). The other category (yellow) represents reads mapping elsewhere in the *S. carpocapsae* genome.

**Supplementary Figure S4**. Differential expression analysis of *S. carpocapsae* miRNAs in response to insect hemolymph *in vitro* treatment. MA-plot showing the absolute and relative expression of all annotated mature miRNAs with at least 3 reads per million in three of the six libraries. The X-axis shows the average counts-per-million across all libraries, in log2 scale. The Y-axis shows fold-change values comparing hemolymph

treatment to control libraries, in log2 scale. Positive log2 fold-changes represent miRNAs with higher expression under haemolymph treatment. Significantly differentially expressed miRNAs (using a False Discovery Rate <0.1) are shown as solid red circles. The two most expressed miRNAs are shown as solid blue circles.

**Supplementary Figure S5**. Functional annotation of differentially expressed proteins induced by insect intestine.

**Supplementary Figure S6**. Functional annotation of differentially expressed proteins induced by insect haemolymph.

**Supplementary Figure S7.** Analysis pipeline for annotation of protein-coding genes in the genome of *S. carpocapsae* strain Breton.

**Supplementary Figure S1. Graphic representation of the abundance of the different protein families (PFAM domains) in 10 different nematode genomes compared with the top 40 abundant families in *S. carpocapsae*.**

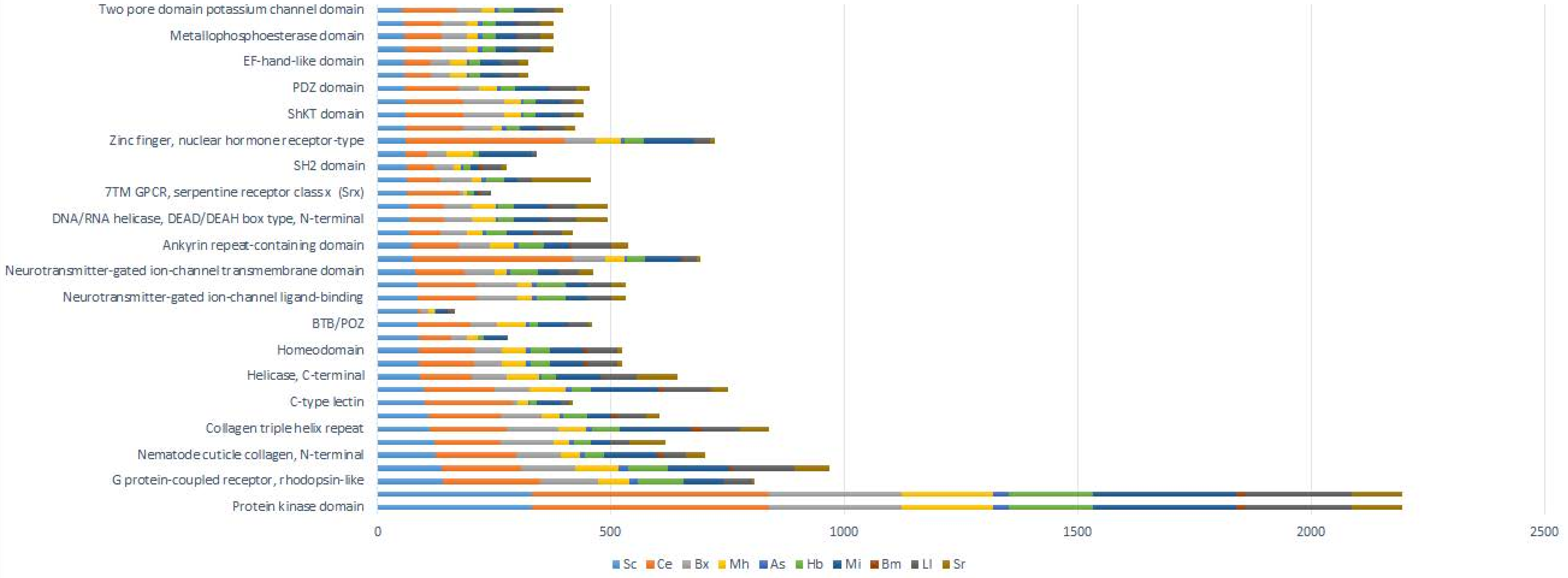

Supplementary Figure S2

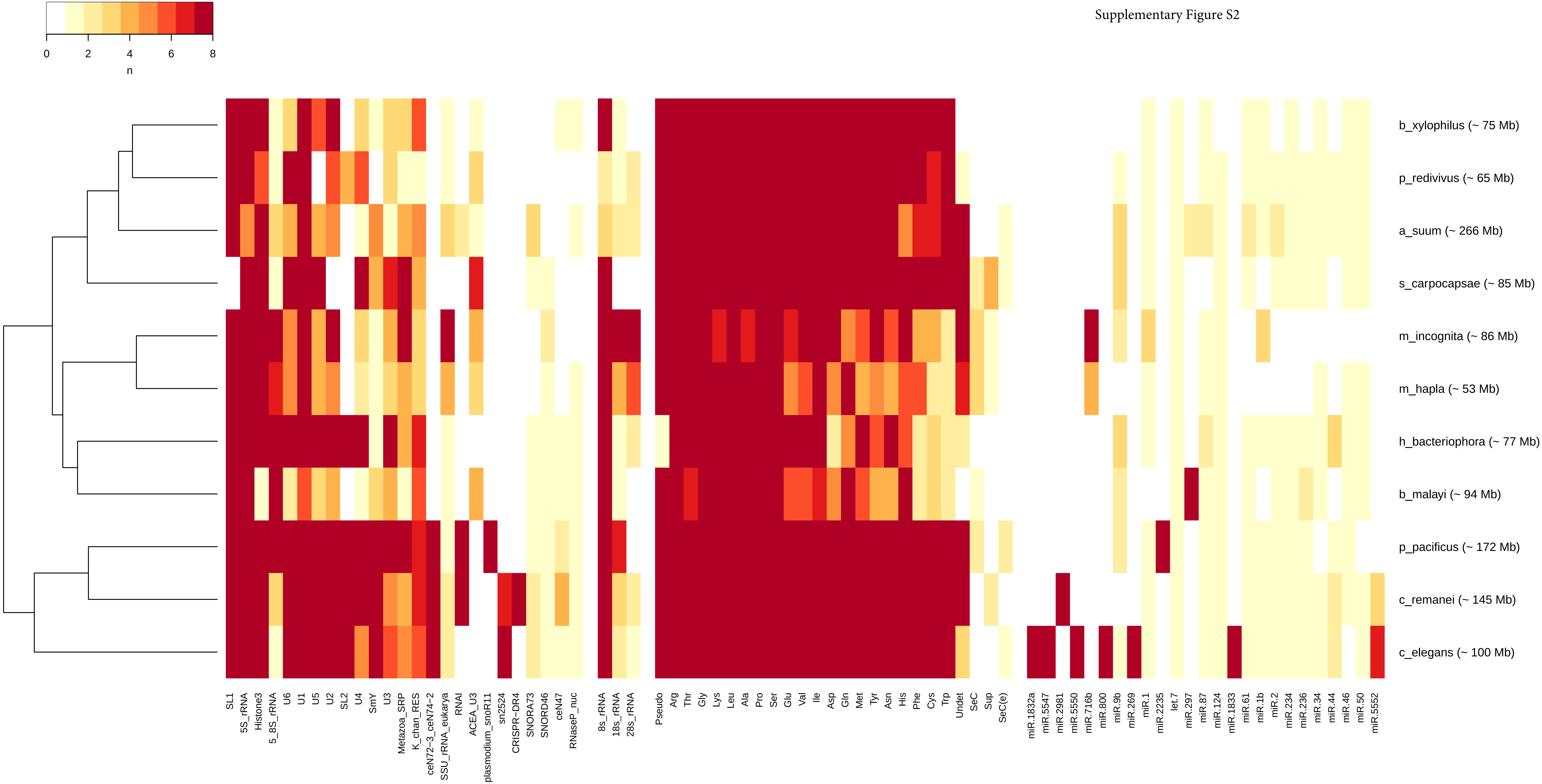

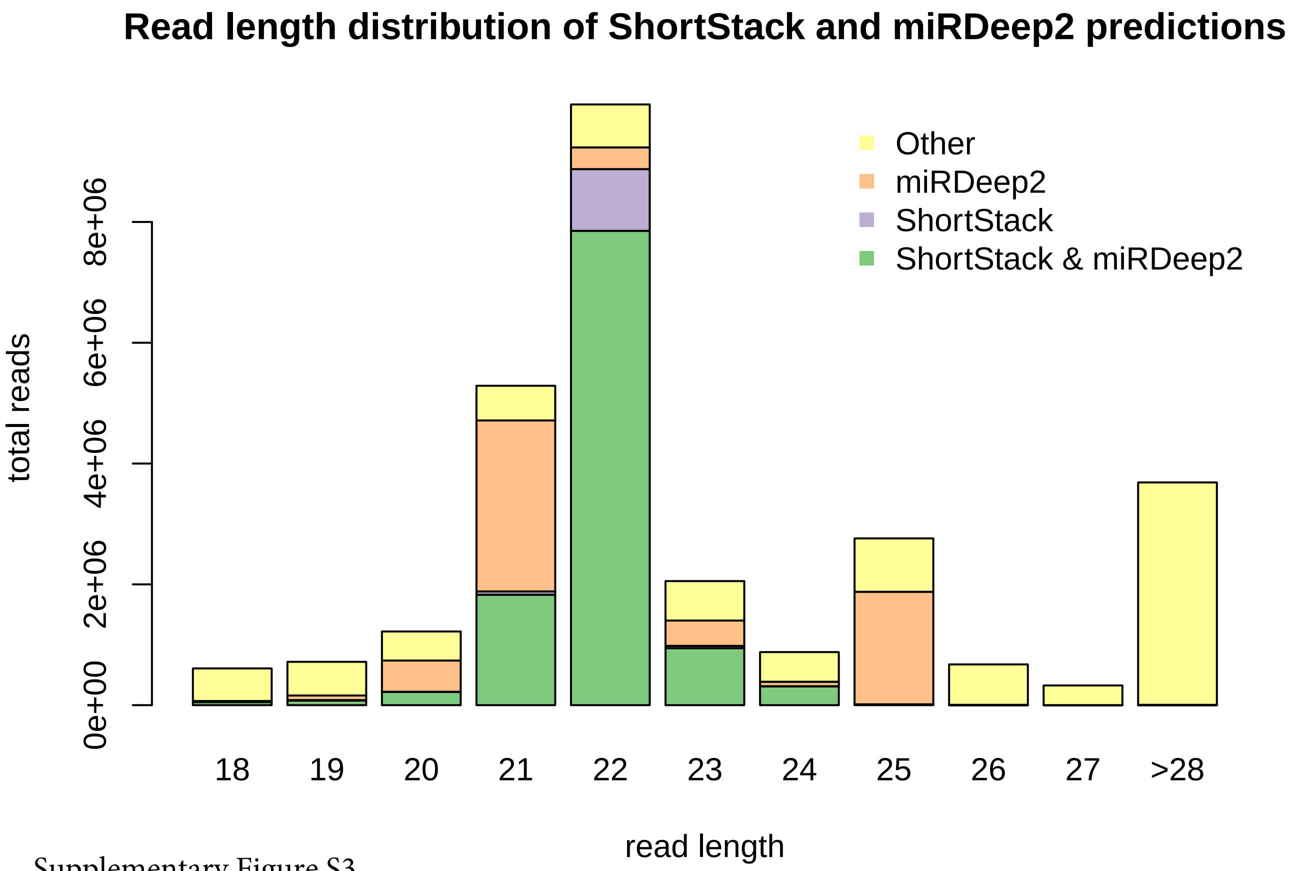

Supplementary Figure S3

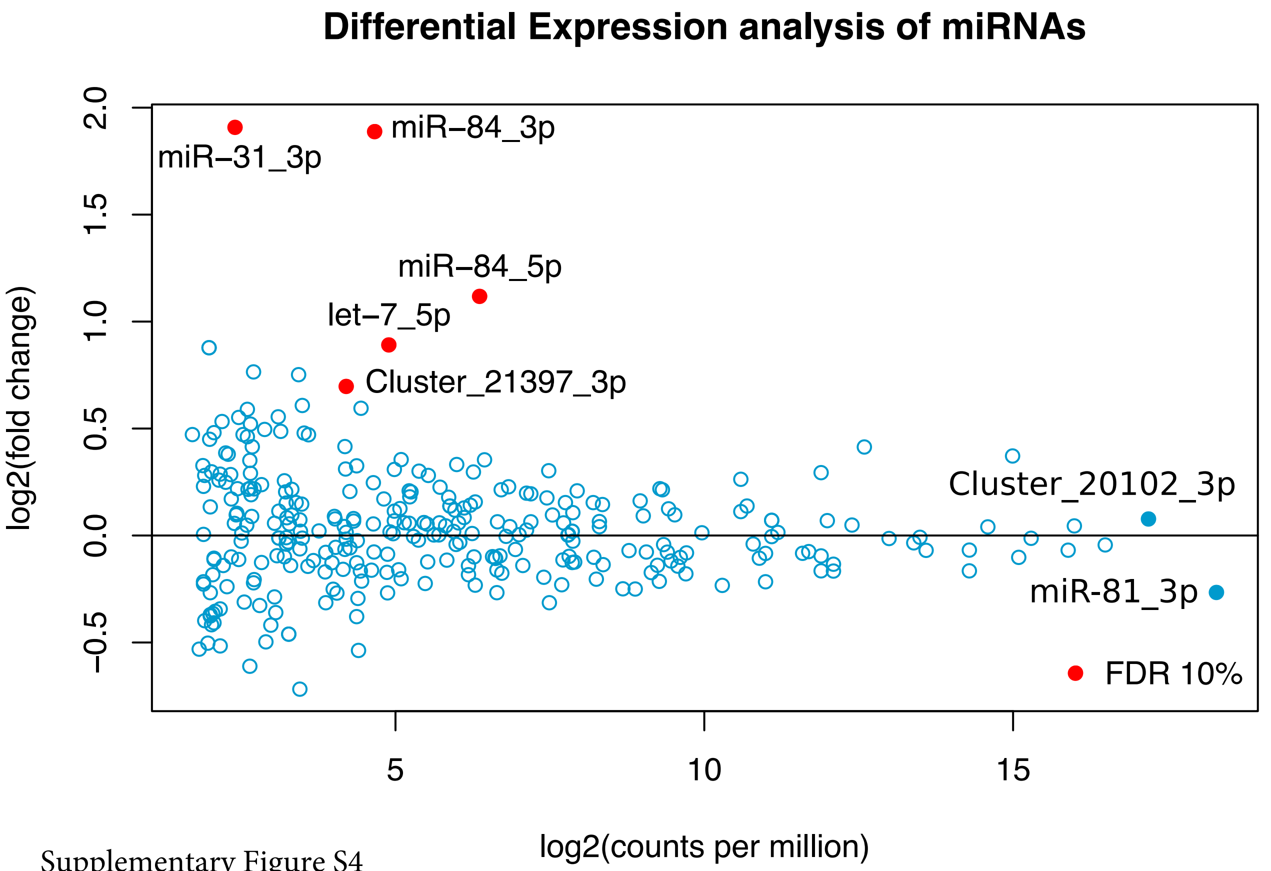


Supplementary Figure S4

# Functional annotation of differential proteins found under insect intestine induction

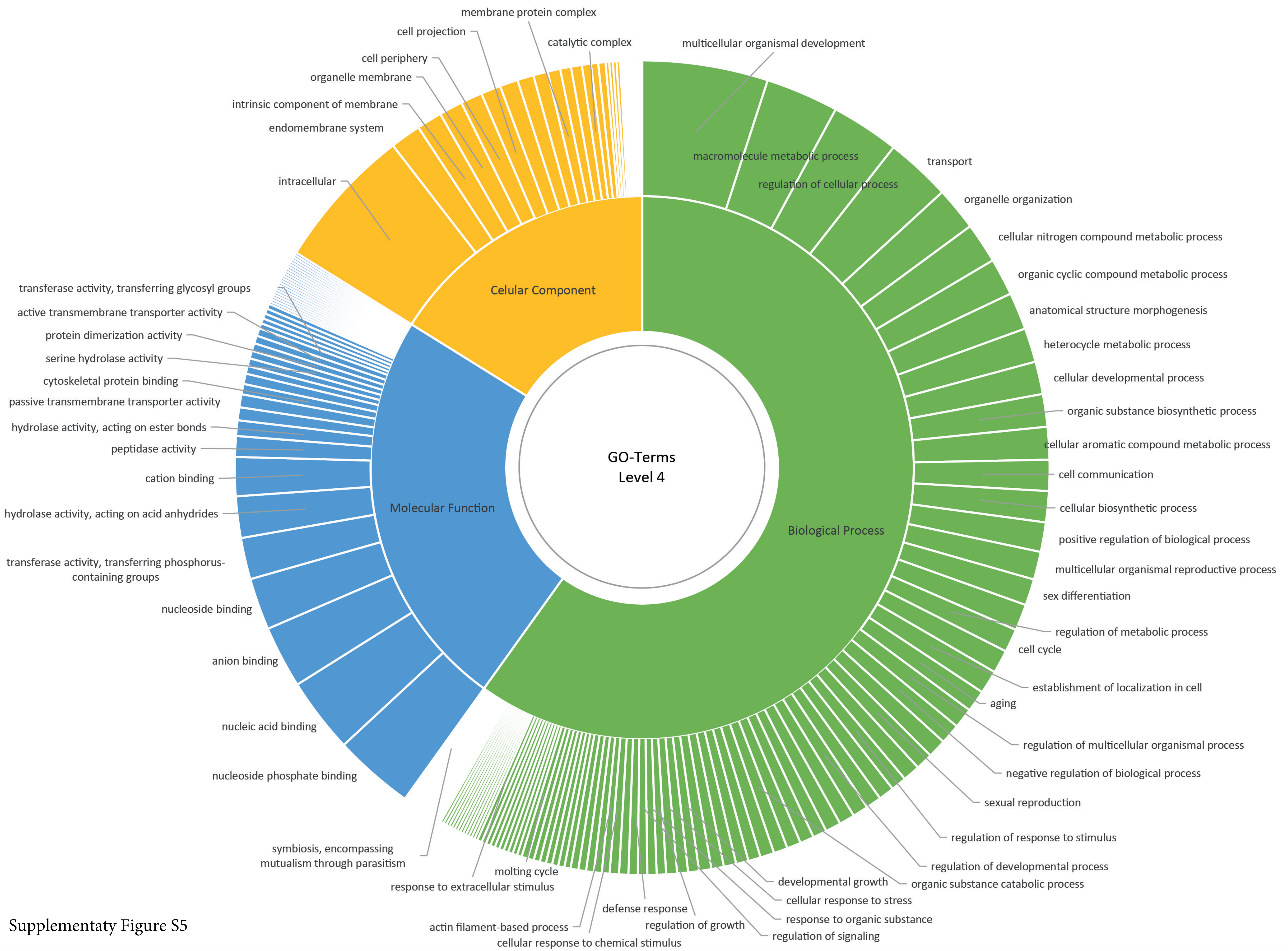


Supplementaty Figure S5

# Functional annotation of differential proteins found under insect haemolymph induction

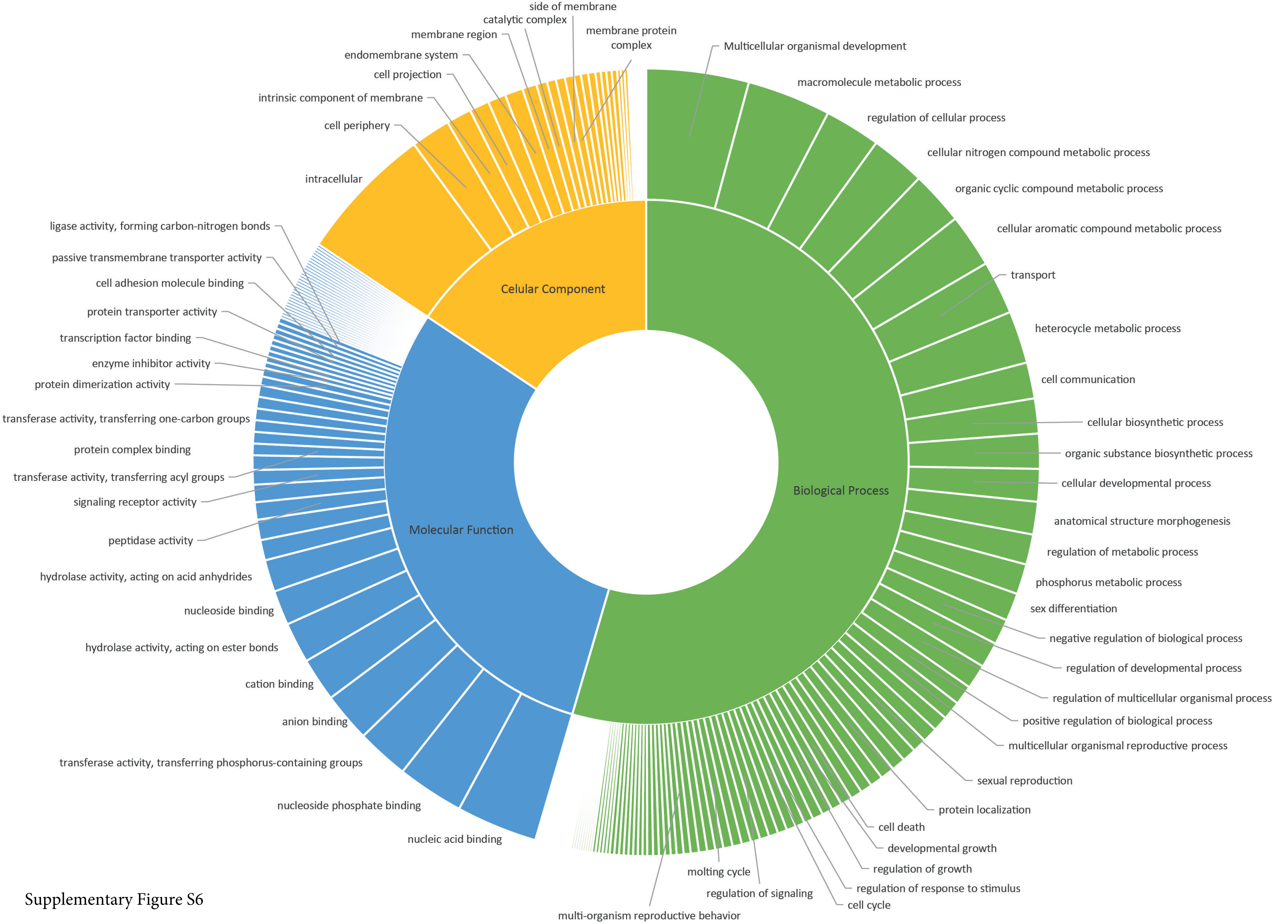


Supplementary Figure S6

Supplementary Figure S7

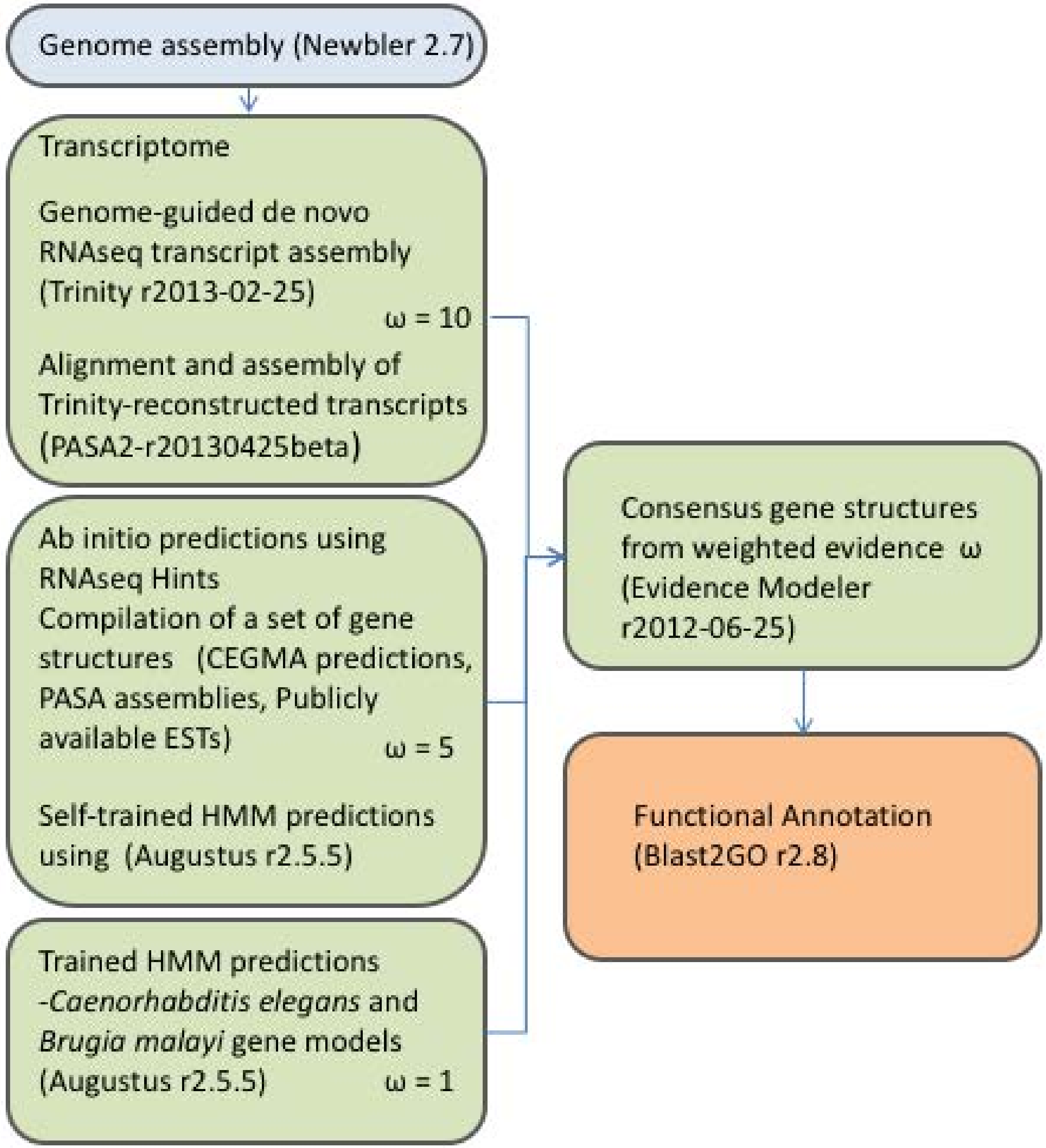

Supplement: Supplementary Figures S1–S7 [file srep37536-s1.pdf]
