# Supplementary material for: The genome, transcriptome, and proteome of the nematode Steinernema carpocapsae: evolutionary signatures of a pathogenic lifestyle
Source: Sci Rep. 2016 Nov 23;6:37536. doi: 10.1038/srep37536 (PMC5120318; doi:10.1038/srep37536)
Supplement: Supplementary Table S1 [file srep37536-s2.doc]

| The genome, transcriptome, and proteome of the nematode *Steinernema carpocapsae*: evolutionary signatures of a pathogenic lifestyle  Rougon-Cardoso A., Flores-Ponce, M., Ramos-Aboites, H.E., Martinez-Guerrero, C.E., Hao, Y-J., Cunha, L., Rodríguez-Martinez J.A., Ovando-Vázquez, C., Bermúdez-Barrientos, J.R., Abreu-Goodger, C., Chavarria-Hernández, N., Simões, N., Montiel, R.  **Supplementary Table S1.** Interspersed repeats in the genome of *Steinernema carpocapsae* strain Breton | | | | | | |
| --- | --- | --- | --- | --- | --- | --- |
| Type^a^ |  | Number | Bases | % of genome | % of total repeats |  |
| **Retrotransposons** | |  |  |  |  |  |
|  | LINE/L1 | 76 | 12,704 | 0.0150 | 0.2149 |  |
|  | LINE/RTE-RTE | 113 | 19,721 | 0.0233 | 0.3336 |  |
|  | LINE/Cr1 | 374 | 102,451 | 0.1213 | 1.7330 |  |
|  | LINE/Jockey | 34 | 4,925 | 0.0058 | 0.0833 |  |
|  |  |  |  |  |  |  |
|  | SINE/tRNA-RTE | 156 | 12,927 | 0.0153 | 0.2187 |  |
|  | SINE other | 432 | 42,508 | 0.0503 | 0.7190 |  |
|  |  |  |  |  |  |  |
|  | LTR/Gypsy | 388 | 47,846 | 0.0566 | 0.8093 |  |
|  | LTR/Pao | 129 | 23,551 | 0.0279 | 0.3984 |  |
|  |  |  |  |  |  |  |
| **DNA transposons** | |  |  |  |  |  |
|  | DNA/MULE-MuDR | 72 | 4,717 | 0.0056 | 0.0798 |  |
|  | DNA/hAT-Ac | 388 | 52,974 | 0.0627 | 0.8961 |  |
|  | DNA/Merlin | 106 | 25,346 | 0.0300 | 0.4287 |  |
|  | DNA/TcMar-Tc1 | 327 | 73,770 | 0.0873 | 1.2479 |  |
|  | DNA/TcMar-Tc4 | 50 | 73,770 | 0.0873 | 1.2479 |  |
|  | DNA/TcMar-m44 | 43 | 8,571 | 0.0101 | 0.1450 |  |
|  | RC/Helitron | 105 | 19,110 | 0.0226 | 0.3233 |  |
|  | DNA other | 111 | 14,638 | 0.0173 | 0.2476 |  |
|  |  |  |  |  |  |  |
|  | snRNA | 114 | 15,311 | 0.0181 | 0.2590 |  |
|  |  |  |  |  |  |  |
| **Low complexity** | | 1,425 | 64,870 | 0.0768 | 1.0973 |  |
| **Simple repeats** | | 5,896 | 281,888 | 0.3337 | 4.7683 |  |
| **Unclassified** | | 27,679 | 5,010,098 | 5.9311 | 84.7489 |  |
| **Total** |  | 38,018 | 5,911,696 | 6.9984 | 100 |  |
| ^a^All repeat types were assigned according to homology to the Repbasedatabase | | | | | | |
| (http://www.girinst.org/repbase). | | |  |  |  |  |
